# A novel prognostic nomogram for patients with extragastric mucosa‐associated lymphoid tissue lymphoma: A multicenter study

**DOI:** 10.1002/cam4.4702

**Published:** 2022-04-29

**Authors:** Xiaoqian Li, Huangming Hong, He Huang, Liqun Zou, Zegeng Chen, Zhihui Zhang, Liling Zhang, Xiaojie Fang, Hongqiang Guo, Ke Xie, Ying Tian, Suxia Lin, Yungchang Chen, Wei Zhang, Yuyi Yao, Fei Pan, Huawei Weng, Tongyu Lin

**Affiliations:** ^1^ Department of Medical Oncology, Sun Yat‐sen University Cancer Center, Guangzhou State Key Laboratory of Oncology in Southern China, and Collaborative Innovation Center of Cancer Medicine Guangzhou China; ^2^ Department of Senior and Phase I Clinical Trial Ward, Sichuan Cancer Hospital & Institute, Sichuan Cancer Center School of Medicine University of Electronic Science & Technology of China, Sichuan, Province Chengdu China; ^3^ Department of Oncology, West China Hospital Sichuan University Chengdu Sichuan China; ^4^ Cancer Centre, Union Hospital, Tongji Medical College Huazhong University of Science and Technology Wuhan China; ^5^ Department of Medical Oncology He Nan Cancer Hospital Zhengzhou China; ^6^ Department of Oncology Sichuan Provincial People's Hospital Chengdu China

**Keywords:** extragastric MALT lymphoma, nomogram, overall survival, prognosis, progression‐free survival

## Abstract

**Background:**

The aim of this study was to explore predictors and construct a nomogram for risk stratification in primary extragastric mucosa‐associated lymphoid tissue (MALT) lymphoma.

**Methods:**

Extragastric MALT lymphoma cases newly diagnosed between November 2010 and April 2020 were assessed to construct a progression‐free survival (PFS)‐related nomogram. We also performed external validation of the nomogram in an independent cohort.

**Results:**

We performed multivariate analyses of 174 patients from 3 hospitals who were included in the training cohort. Stage, hepatitis B virus surface antigen (HBsAg) status, and Ki67 expression were significantly associated with PFS. These three factors were used to construct a nomogram, which was shown to have a C‐index of 0.89. Two risk groups (low risk and high risk) were identified by the prognostic model. The 5‐year PFS was 98.9% for the low‐risk group and 69.3% for the high‐risk group (*p* < 0.001). The overall survival (OS) could also be effectively distinguished by the nomogram, resulting in an OS of 100% for the low‐risk group and 94.6% for the high‐risk group (*p* = 0.01). These results were validated and confirmed in an independent cohort with 165 patients from another three hospitals. The 5‐year PFS rates were 94.8% and 66.7% for the low‐risk and high‐risk groups, respectively (*p* < 0.001). The 5‐year OS rates were 97.9% and 88.4%, respectively (*p* = 0.016).

**Conclusion:**

The nomogram could well distinguish the prognosis of low‐ and high‐risk patients with extragastric MALT lymphoma and is thus recommended for clinical use.

## Graphical abstract

Extragastric MALT lymphoma is a rare subtype of NHL lymphoma, and without specific prognostic model. The prognostic model was constructed from the largest cohort of extragastric MALT lymphoma cases and it was the first study to establish a prognostic model in extragastric MALT lymphoma. The novel nomogram index could stratify extragastric MALT lymphoma patients into two risk groups which have significantly different prognosis, both in PFS and OS.

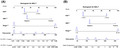

## 1 INTRODUCTION

Extranodal marginal zone B cell lymphoma (MZL) of mucosa‐associated lymphoid tissue (MALT lymphoma) is a unique subtype of non‐Hodgkin lymphoma (NHL) defined by the World Health Organization (WHO) classification of lymphoid malignancies that accounts for 7%–8% of newly diagnosed lymphomas.^1^ MALT lymphoma can be divided into two types: gastric and extragastric. The stomach is the most common primary site, with cases in the stomach accounting for 50% of MALT lymphoma cases.^2^ Common extragastric types of MALT lymphoma are lung, head and neck, and orbital MALT lymphoma, while cases involving intestine, liver, thyroid, and breast are rarer.^3^, ^4^ Patients with MALT lymphoma usually experienced an indolent course and good outcomes.^5^, ^6^, ^7^

Therapeutic choice varies by the primarily involved organ and the extent of disease. For gastric MALT lymphoma, two‐thirds of patients have chronic Helicobacter pylori (HP) infection. Approximately 75%–80% of patients will achieve regression after HP eradication treatment.^2^ Patients with the t(11;18)(q21;q21)/API2‐MALT1 translocation are resistant to antibiotics, and thus, this translocation can be used to predict the response to antibiotic regimens.^8^ For non‐gastric MALT lymphoma patients, surgery, radiotherapy, rituximab alone, or in combination with chemotherapy, and “chemo‐free” approaches, including lenalidomide, have been effective treatment options.^9^ After first‐line treatment, most patients have an excellent prognosis, with 5‐year overall survival (OS) rates higher than 90% and a 10‐year survival rate of 75%–80%.^10^, ^11^ However, recurrences have repeatedly been reported in patients with MALT lymphoma after a prolonged follow‐up time,^12^, ^13^ and there are few specific risk models for predicting recurrence.

The prognostic index of MZL (MZLPI) is a useful prognostic index for non‐gastric MZL, but it is not suitable for the unique MALT subtype.^14^ The MALT‐International Prognostic Index (IPI),^15^ which considers stage III‐IV disease, age >70 years, and elevated lactate dehydrogenase (LDH) levels, has been widely used in clinical practice as a prognostic indicator in MALT lymphoma patients.^16^, ^17^ This model covers both gastric and extragastric MALT lymphoma. However, the two subtypes have different treatments and prognoses.^18^, ^19^ Here, we constructed a specific nomogram with an internal cohort for extragastric MALT lymphoma to distinguish high‐ and low‐risk patients and guide the selection of treatment and externally validated it.

## 2 PATIENTS AND METHODS

### 2.1 Patients

From November 2010 to April 2020, patients diagnosed with extragastric MALT lymphoma at three cancer centers (Wuhan Union Hospital, He Nan Cancer Hospital, and Sichuan Provincial People's Hospital) were screened as the training cohort for nomogram construction. The validation cohort was recruited from another three hospitals (Sun Yat‐sen University Cancer Center, West China Hospital, and Sichuan Cancer Hospital & Institute). The inclusion criteria were as follows: histologically proven MALT lymphoma according to the WHO Classification of Tumors of Hematopoietic and Lymphoid Tissue^20^ and complete follow‐up data and clinical information. Patients lacking clinical data or who were diagnosed with primary gastric or splenic MZL were excluded. The primary sites of extra‐gastric MALT lymphoma in our study include orbital, thyroid, salivary gland, lung, mediastinal, soft tissue. The “other” included upper airways, breast, intestinal sites, liver, tonsil, tongue, colon, and skin which were rare sites, it also included advanced MALT lymphoma which was extensively invaded and cannot be identified as the primary site. This study was approved by the ethics committee of Sun Yat‐sen University Cancer Center. The project was also approved by the institutional review board of each participating institution.

### 2.2 Clinical indicators and outcomes

We collected data regarding the following clinical features: sex, age, Ann Arbor stage, Eastern Cooperative Oncology Group performance status (ECOG PS), lactate dehydrogenase (LDH), β2‐microglobulin, erythrocyte sedimentation rate (ESR), platelet (PLT), hemoglobin (Hb), lymphocytes, neutrophils, monocytes, neutrophil‐to‐lymphocyte ratio (NLR), lymphocyte‐to‐monocyte ratio (LMR), IPI, MALT‐IPI, Ki67 level, extranodal involvement status, and hepatitis B virus surface antigen (HBsAg) status at diagnosis. Other clinical indicators included the curative effect of the first‐line treatment and treatment type information (radiotherapy, surgery, or immunotherapy). The indicators above were included according to prognostic indicators recommended in the guidelines or reported in previous studies. The NLR was defined as the ratio of the absolute neutrophil count (ANC) to the absolute lymphocyte count (ALC). The LMR was the ratio between the ALC and the monocyte count. Progression‐free survival (PFS) was defined as the time from diagnosis to disease progression, relapse, or death from any cause, with censoring at the time of the last follow‐up. OS was defined as the time from diagnosis to death from any cause, with censoring at the time of the last follow‐up.

### 2.3 Nomogram construction and statistical analysis

In the design of the nomogram, univariate analysis was applied to identify prognostic indicators. These factors included all the factors mentioned above. Each factor was defined as binary/categorical type and input. Kaplan–Meier analysis was used to check the proportional hazard assumption required for Cox regression. PFS was used as the endpoint in the construction of the nomogram model. The chained equations approach was used to input the data. Missing values are unavoidable in retrospective studies. For missing values, multiple imputation method was used. The method had been used in a previous study.^21^ Multivariate Cox proportional hazards models were also used to identify independent prognostic indicators, which were then employed to develop the nomogram. All significant factors in the univariate analysis were entered into the multivariate analysis. Receiver operating characteristic (ROC) curves were constructed for the internal cohort and for external validation. Time‐dependent ROC curve was used for PFS in different time points.^22^ The concordance index (C index) was estimated from the area under the ROC curve (AUC), and a calibration plot was used to assess the agreement between the predicted and observed survival probabilities in the internal cohort. For the validation cohort, each patient received a risk score according to the nomogram, which was used for classification. The optimal cut‐off value for continuous variables was also determined by ROC curve analysis. Chi‐squared test was used to compare the difference of clinical characteristics between training cohort and external cohort. Survival analysis of the different cohorts was performed using Kaplan–Meier curves and the log‐rank test. All reported *p*‐values were two‐sided, and *p* < 0.05 was considered to indicate statistical significance. Data analysis was performed with R version 4.0.2 via the survival and design packages.

## 3 RESULTS

### 3.1 Clinical characteristics

In the training cohort, 174 patients were enrolled. The median age was 65 years, ranging from 18 to 80 years; 80 (46%) patients were male, and 94 (54%) were female. A majority of the patients (85.6%, 149/174) had a good ECOG PS score of 0–1. Sixty‐seven patients (38%) had stage III or IV disease. Most patients (79.3%, 138/174) were at low risk or low intermediate risk according to the IPI, while 99 patients (56.9%) were at low risk according to the MALT‐IPI. Sixty‐eight (39.1%) patients had involvement of ≥2 extranodal sites. Thirty‐two patients (13.2%) had HBsAg positivity. A total of 107 (61.5%) patients achieved complete remission (CR) after first‐line treatment. Among all patients, 101 (58%) received surgery, 55 (31.6%) received immunotherapy, and 80 (46%) received radiotherapy. The details of the training cohort are shown in Table 1. A total of 165 patients from another three cancer centers were included to validate the prognostic model, and the characteristics of those patients are also shown in Table 1.

**TABLE 1:** Clinical characteristics of patients

Characteristics	Training cohort (%) (*n* = 174)	External validation cohort (%) (*n* = 165)	*p* value
Age			
<60	109 (62.6)	106 (64.2)	0.205
≥60	65 (37.4)	59 (35.8)	
Sex			
Male	80 (46)	84 (50.9)	0.690
Female	94 (54)	81 (49.1)	
ECOG PS			
0–1	149 (85.6)	143 (86.7)	0.200
≥2	25 (14.4)	22 (13.3)	
B symptom			
No	171 (98.3)	163 (98.8)	0.421
Yes	3 (1.7)	2 (1.2)	
Ann Arbor stage			
I–II	107 (61.5)	126 (76.4)	0.060
III–IV	67 (38.5)	39 (23.6)	
Subtype			
Orbital	53 (30.5)	43 (26.1)	0.659
Thyroid	13 (7.5)	12 (7.3)	
Salivary gland	27 (15.5)	24 (14.5)	
Lung	21 (12.1)	14 (8.5)	
Mediastinal	5 (2.8)	6 (3.6)	
Soft tissue	7 (4.0)	6 (3.4)	
Other	48 (27.6)	60 (36.6)	
β2‐microglobulin			
Normal	127 (72.9)	126 (76.3)	0.420
Elevated	47 (26.1)	39 (23.7)	
LDH			
Normal	135 (77.6)	130 (78.8)	0.070
Elevated	39 (22.4)	35 (21.2)	
IPI			
0–2	138 (79.3)	134 (81.2)	0.684
≥3	36 (20.7)	31 (18.8)	
MALT‐IPI			
0	99 (56.9)	110 (66.7)	0.074
≥1	75 (43.1)	55 (33.3)	
Extranodal sites involved			
0–1	106 (60.9)	101 (61.2)	0.070
≥2	68 (39.1)	64 (38.8)	
HBsAg status			
Positive	32 (13.2)	28 (17)	0.313
Negative	142 (86.8)	137 (83)	
Hemoglobin			
≥120 g/L	122 (70.1)	106 (64.2)	0.761
<120 g/L	52 (29.9)	59 (35.8)	
LMR			
≥2	92 (52.9)	84 (50.9)	0.210
<2	82 (47.1)	81 (49.1)	
NLR			
≥6.10	41 (23.6)	39 (21.8)	0.420
<6.10	133 (76.4)	126 (78.2)	
Curative effect			
CR	107 (61.5)	117 (70.9)	0.183
PR	36 (20.7)	31 (18.8)	
SD	22 (12.6)	11 (6.7)	
PD	9 (5.2)	6 (3.6)	
Surgery			
Yes	101 (58)	105 (63.6)	0.317
No	73 (42)	60 (36.4)	
Immunotherapy			
Yes	55 (31.6)	60 (36.4)	0.361
No	119 (68.4)	105 (63.6)	
Radiotherapy			
Yes	80 (46)	84 (51)	0.385
No	94 (54)	81 (49)	

Abbreviations: ECOG PS, Eastern Cooperative Oncology Group performance status; LDH, lactic dehydrogenase; IPI, International Prognostic Index; MALT‐IPI, MALT‐International Prognostic Index; HBsAg, Hepatitis B virus surface antigen; LMR, lymphocyte‐to‐monocyte ratio; NLR, neutrophil‐to‐lymphocyte ratio; CR, complete remission; PR, partial remission; SD, stable disease; PD, progressive disease.

### 3.2 Univariate and multivariate Cox regression analyses

According to the ROC curve for PFS, the NLR cut‐off was 6.0, which provided an AUC of 0.749 (95% confidence interval CI, 0.627–0.870, *p* < 0.05); the LMR cut‐off was 2.1, which provided an AUC of 0.704 (95% CI, 0.586–0.822, *p* < 0.05); and the Ki67 cut‐off was 10%, which provided an AUC of 0.7 (95% CI, 0.595–0.805, *p* < 0.05). In the univariate analysis, the following variables were statistically significant in predicting recurrence: stage, ECOG PS, radiotherapy, NLR, IPI, MALT‐IPI, Ki67 level, HBsAg status, and curative effect. In the multivariate analysis, three independent factors were found: stage (95% CI, 6.846–76.588, *p* < 0.001), HBsAg status (95% CI, 2.243–11.820, *p* < 0.001) and Ki67 level (95% CI, 2.062–11.386, *p* < 0.001) (Table 2).

**TABLE 2:** Univariate and multivariate analyses of prognostic factors for PFS

	Univariate			Multivariate		
	HR	95% CI	*p* value	HR	95% CI	*p* value
Age (male vs female)	0.89	0.81–4.41	0.136	—	—	—
Sex (≥60 vs <60)	0.75	0.32–1.78	0.514	—	—	—
ECOG PS (≥2 vs 0–1)	8.27	3.95–17.29	<0.001	2.295	0.585–9.00	0.233
Stage (III‐IV vs I‐II)	22.89	6.84–76.58	<0.001	3.887	3.03–20.67	<0.001
β2‐microglobulin (elevated vs normal)	1.93	0.83–4.47	0.123	—	—	—
LDH (elevated vs normal)	1.89	0.73–3.98	0.055	—	—	—
IPI (≥3 vs 0–2)	2.82	1.32–6.04	0.007	0.713	0.21–2.45	0.591
MALT‐IPI (≥1 vs 0)	5.22	2.25–12.07	<0.001	0.414	0.11–1.60	0.202
Ki67 level (≥10% vs <10%)	4.85	2.06–11.38	<0.001	2.14	1.95–2.59	0.032
HBsAg status (positive vs negative)	5.15	2.24–11.82	<0.001	3.74	1.70–5.49	<0.001
Hemoglobin (<120 g/L vs ≥120 g/L)	1.47	0.64–3.44	0.368	—	—	—
First‐line therapy efficacy (SD/PD vs CR/PR)	6.40	2.94–13.93	<0.001	1.04	0.33–3.3	0.944
Radiotherapy (yes vs no)	0.43	0.19–0.99	0.048	0.86	0.32–2.37	0.780
LMR (≥2 vs <2)						
NLR (≥6 vs < 6)	3.02	1.44–6.31	0.003	0.83	0.25–2.71	0.756
Surgery (yes vs no)	0.58	0.25–1.37	0.215			
Immunotherapy (yes vs no)	0.42	0.14–2.9	0.332	—	—	—

Abbreviations: CR, complete remission; ECOG PS, Eastern Cooperative Oncology Group performance status; HBsAg, Hepatitis B virus surface antigen; IPI, International Prognostic Index; LDH, lactic dehydrogenase; LMR, lymphocyte‐to‐monocyte ratio; MALT‐IPI, MALT‐International Prognostic Index; NLR, neutrophil‐to‐lymphocyte ratio; PD, progressive disease; PR, partial remission; SD, stable disease; PD, progressive disease.

### 3.3 Nomogram construction and validation

**FIGURE 1:**
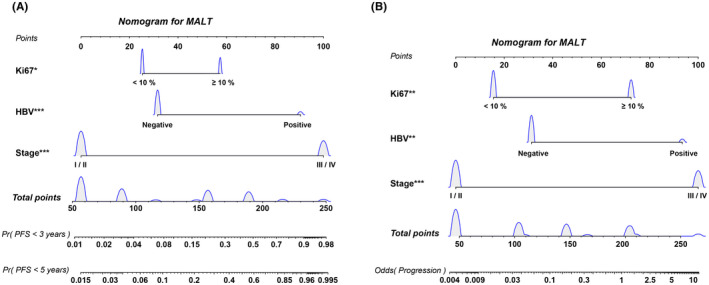
(A) Nomogram for predicting the 3‐year and 5‐year PFS probability for patients with extragastric MALT lymphoma. (B) Nomogram for predicting the probability of recurrence

**FIGURE 2:**
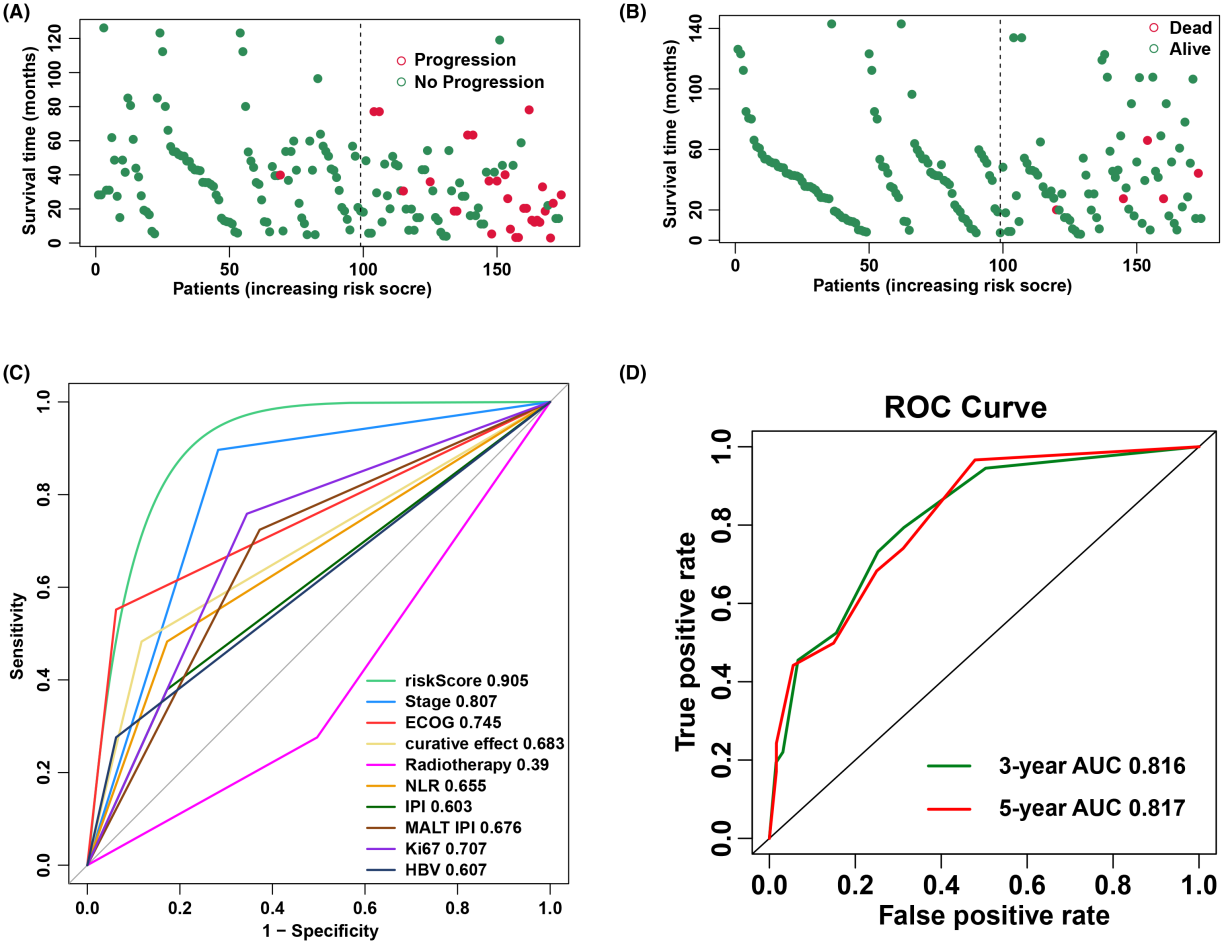
(A) Distribution of recurrence in the two groups; (B) Distribution of death in the two groups. (C) Sensitivity and specificity comparison between the novel nomogram and other factors in the training cohort. (D) Progression‐free survival probability curves of the nomogram training cohort according to the two risk groups defined by the nomogram

The three independent prognostic parameters identified in the multivariate analysis were used for nomogram construction (Figure 1A,B). The C index (0.89) was calculated to assess the validity and reliability of the developed nomogram in the internal cohort. Based on the developed nomogram, two discrete risk groups were determined by the total points: a low‐risk group and a high‐risk group (Figure S1). A number of events were more likely to occur in the high‐risk group (Figure 2A,B). According to ROC curve analysis, the new risk score system had better accuracy than the IPI (0.603), MALT‐IPI (0.676), and other single prognostic factors (Figure 2C). The predictive accuracy for 3‐year PFS and 5‐year PFS was measured by calculating the C‐index: 0.816 and 0.817, respectively (Figure 2D). The new nomogram also had better sensitivity and specificity than single factors in the validation cohort, and the AUC was 0.905 (95% CI, 0.749–0.959) (Figure S2).

### 3.4 Survival prediction with the internal and validation cohorts

**FIGURE 3:**
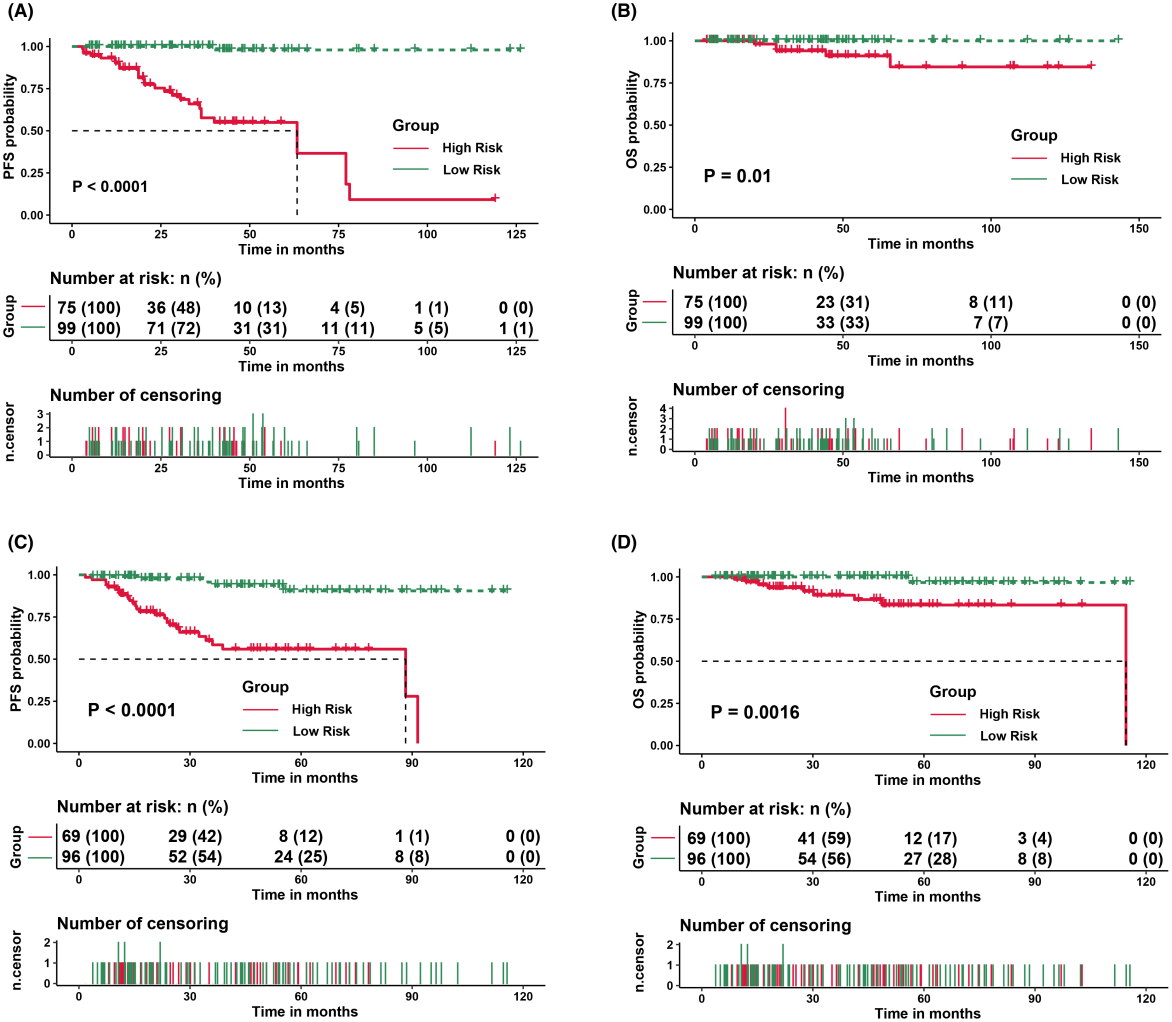
Progression‐free survival (PFS) and overall survival (OS) for patients with extragastric MALT lymphoma defined by the nomogram. (A and B) Training cohort patients. (C and D) Validation cohort patients

The prognosis between the low‐risk and high‐risk groups was significantly different, with a 5‐year PFS of 98.9% versus 69.3% (*p* < 0.001) and a 5‐year OS of 100% versus 98.9% (*p* = 0.01) (Figure 3A,B). The validation cohort showed good agreement with the internal cohort. Two risk groups could be categorized using the nomogram, and the 5‐year PFS rates were 94.8% and 66.7% in the low‐risk and high‐risk groups, respectively (*p* < 0.001). The 5‐year OS was 97.9% versus 88.4% (*p* = 0.0016) (Figure 3C,D).

**FIGURE 4:**
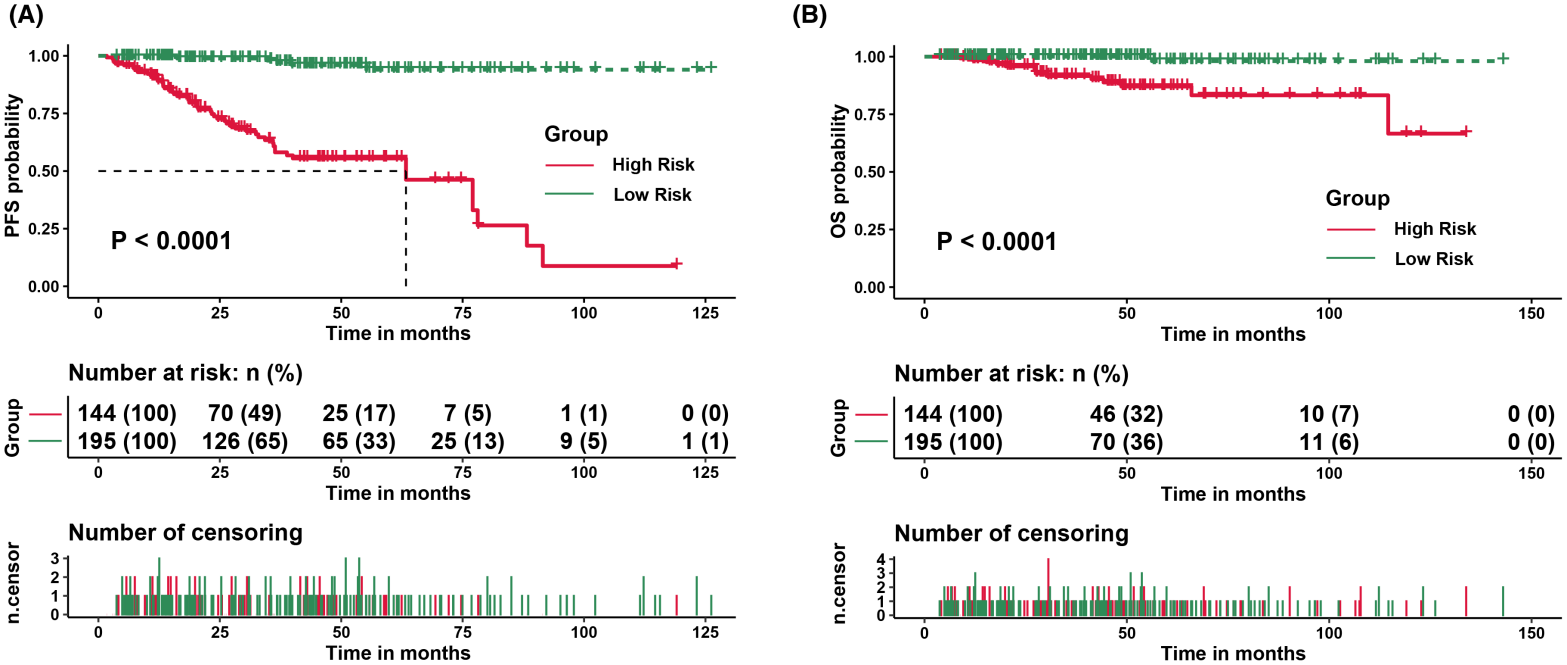
Progression‐free survival (PFS) and overall survival (OS) for all patients

Further analysis of the overall cohort, including both the training cohort and validation cohort, also showed that the low‐risk group had better PFS and OS (Figure 4A,B). We then compared PFS and OS between groups categorized according to the MZLPI and MALT‐IPI. In the PFS analysis, the MZLPI was able to discriminate against each risk group (*p* < 0.001). However, the MALT‐IPI was unable to discriminate the high‐ and intermediate‐risk groups (Figure S3). In the OS analysis, neither the MZLPI nor the MALT‐IPI had the power to discriminate between the high‐ and intermediate‐risk groups (Figure S3).

## 4 DISCUSSION

This was the first study to establish a prognostic model in extragastric MALT lymphoma. To our knowledge, this model was constructed from the largest cohort of extragastric MALT lymphoma cases. Stage, Ki67 level, and HBsAg status were assessed and used to construct the nomogram, which has a good C‐index compared with the IPI and MALT‐IPI. Patients were stratified into two risk groups, the low‐risk group, and the high‐risk group, based on the nomogram. After prognostic analysis, we found that the nomogram had good reliability in determining extragastric MALT prognosis and patient stratification in both the internal and external cohorts.

Recently, other prognostic indices for MALT lymphoma have also been reported. A Korean retrospective study reported a prognostic index for MZL patients. This prognostic index included all MZL subtypes (extranodal, nodal, and splenic); however, these subtypes have nonuniform treatment, and thus, the model is not suitable for MALT lymphoma.^14^ Although another study published that a prognostic model based on elevated serum β2‐microglobulin levels, male sex, and B symptoms could divide MZL patients into different risk groups, it also was unable to categorize extragastric MALT lymphoma as a separate disease.^23^ The MALT‐IPI, which considers stage III‐IV disease, age >70 years and elevated LDH levels, has been widely used in the clinic, but the study population used to construct the MALT‐IPI also included two clinical subgroups (gastric and non‐gastric primary presentation).^15^ Compared with gastric MALT lymphoma, extragastric MALT lymphoma has different clinical characteristics, regimens, and prognoses. Our nomogram was specifically designed for extragastric MALT lymphoma and was more accurate than existing prognostic models in evaluating the prognosis of extragastric MALT lymphoma.

Staging considers disease location and extent and thus suggests prognostic information and provides baseline data. Stage is an independent factor for many subtypes of lymphoma that is being increasingly used in prognostic indices for pretreatment risk stratification and selection of therapy. Patients with early‐stage disease have a better prognosis and lower recurrence rate than those with advanced‐stage disease. The same result was obtained in our study. It has been proposed that hepatitis B virus (HBV) may be an etiologic agent of NHL, especially in B cell lymphoma, which has an HBV infection rate of 8.5%–30.2%.^24^, ^25^, ^26^, ^27^ HBV infection is a global health problem. HBsAg prevalence was 3.61% worldwide. Compared to the with highly endemic HBV (≥8%), the prevalence of HBV infection in China was 5.49% which belongs to a moderate level.^28^ Several studies have suggested that HBV may act as an etiologic factor in NHL, especially B cell NHL.^25^, ^29^, ^30^ Previous studies showed that the rate of HBsAg positive in NHL was ranged from 12.85% to 27%.^26^, ^27^, ^31^ In this study, the rates of HBsAg positive rate were 13.2% in the training cohort and 17% in the validation cohort which was similar to the rate in NHL in the previous study. Several studies have indicated that HBsAg positivity is significantly correlated with patient OS and PFS in NHL and that chronic HBV infection increases the risk of NHL (HR 1.74, 95% CI 1.45–2.09).^31^, ^32^, ^33^ The mechanism by which HBV induces lymphomagenesis is postulated to involve chronic stimulation of B cells in the setting of ongoing liver infection. Another study showed that patients with B cell NHL who were infected with HBV had significantly earlier disease occurrence than those who were not infected with HBV.^27^ Consistent with previous studies, we also found that HBV infection was an independent risk factor for prognosis. Ki67 is a nuclear protein synthesized as a cell begins proliferation, and the Ki67 level has been proposed as a quantitative and independent indicator of disease outcome in B cell lymphoma. Previous studies have reported that the Ki67 level can be used to predict prognosis, and a low Ki‐67 level was related to a subgroup of patients with MZL with an excellent prognosis.^34^, ^35^, ^36^ The Ki67 level is a prognostic factor for B cell lymphoma, and the Ki67 range, and mean Ki67 percentage are different in low‐ and high‐grade lymphomas.^37^, ^38^ In the study by Petit B et al., Ki67 >5% indicated significant expression, and >20% indicated increased expression; most MZL cases had lower Ki67 expression than non‐MZL cases.^36^ According to previous studies, there is no specific cut‐off value for MALT lymphoma. In this study, we obtained a cut‐off of 10% for Ki67 expression according to ROC curve analysis. We also found that Ki67 level (with a cut‐off of 10%) was an important predictor of outcome.

For extragastric MALT lymphoma, the incidence of death is low. Patients who suffer relapse can still have long‐term survival after second‐line treatment. According to this, PFS is a good alternative endpoint. Some studies for indolent lymphoma also use PFS as the endpoint.^39^, ^40^, ^41^ Our new nomogram efficiently discriminated patients in the low‐risk group and high‐risk group based on PFS and proved to be valuable with respect to stratifying OS. There are also several limitations in this study. First, this was a retrospective study and thus inevitably has selection bias. Second, this study lacks prognostic molecular indicators. Nevertheless, the data analyzed in this study were obtained from a patient cohort that was treated in a clinical setting, as such, the real‐world were used to construct the nomogram, providing reasonable confidence about its reliability. External validation was used to confirm the feasibility of our nomogram. Future studies should be designed to incorporate more novel biomarkers for better risk stratification and risk‐adapted treatment.

## 5 CONCLUSION

This study clarified the different outcomes of extragastric MALT lymphoma in terms of PFS and OS using a prognostic nomogram. The nomogram showed a good level of discrimination and can provide an individual estimation of risk.

## CONFLICT OF INTEREST

The authors declare that they have no conflicts of interest.

## AUTHOR CONTRIBUTION

Design the study, Tongyu Lin and Xiaoqian Li; methodology, Xiaoqian Li and He Huang; analysis, Xiaoqian Li and Huangming Hong; investigation, Xiaojie Fang, Hongqiang Guo, Ke Xie, Ying Tian, Suxia Lin, Yungchang Chen, Wei Zhang, Yuyi Yao; data curation, Xiaoqian Li, Zegeng Chen, Fei Pan, and Huawei Weng; writing‐original draft preparation, Xiaoqian Li; writing‐review and editing, Xiaoqian Li, Huangming Hong, and Liqun Zou; supervision, Zhihui Zhang and Liling Zhang. All authors have read and agreed to the published version of the manuscript.

## ETHICS STATEMENT

This study was approved by the ethics committee of Sun Yat‐sen University Cancer Center. This study was conducted in accordance with the principles of the Declaration of Helsinki. We did not obtain formal consent from the patients because of the nature of the retrospective observational study.

## Supporting information


Figure S1

Figure S2

Figure S3

## Data Availability

Data sharing is not applicable to this article as no new data were created or analyzed in this study.
