# Supplementary figures and images for: A novel prognostic nomogram for patients with extragastric mucosa‐associated lymphoid tissue lymphoma: A multicenter study
Source: Cancer Med. 2022 Apr 29;11(18):3407–16. doi: 10.1002/cam4.4702 (PMC9487880; doi:10.1002/cam4.4702)

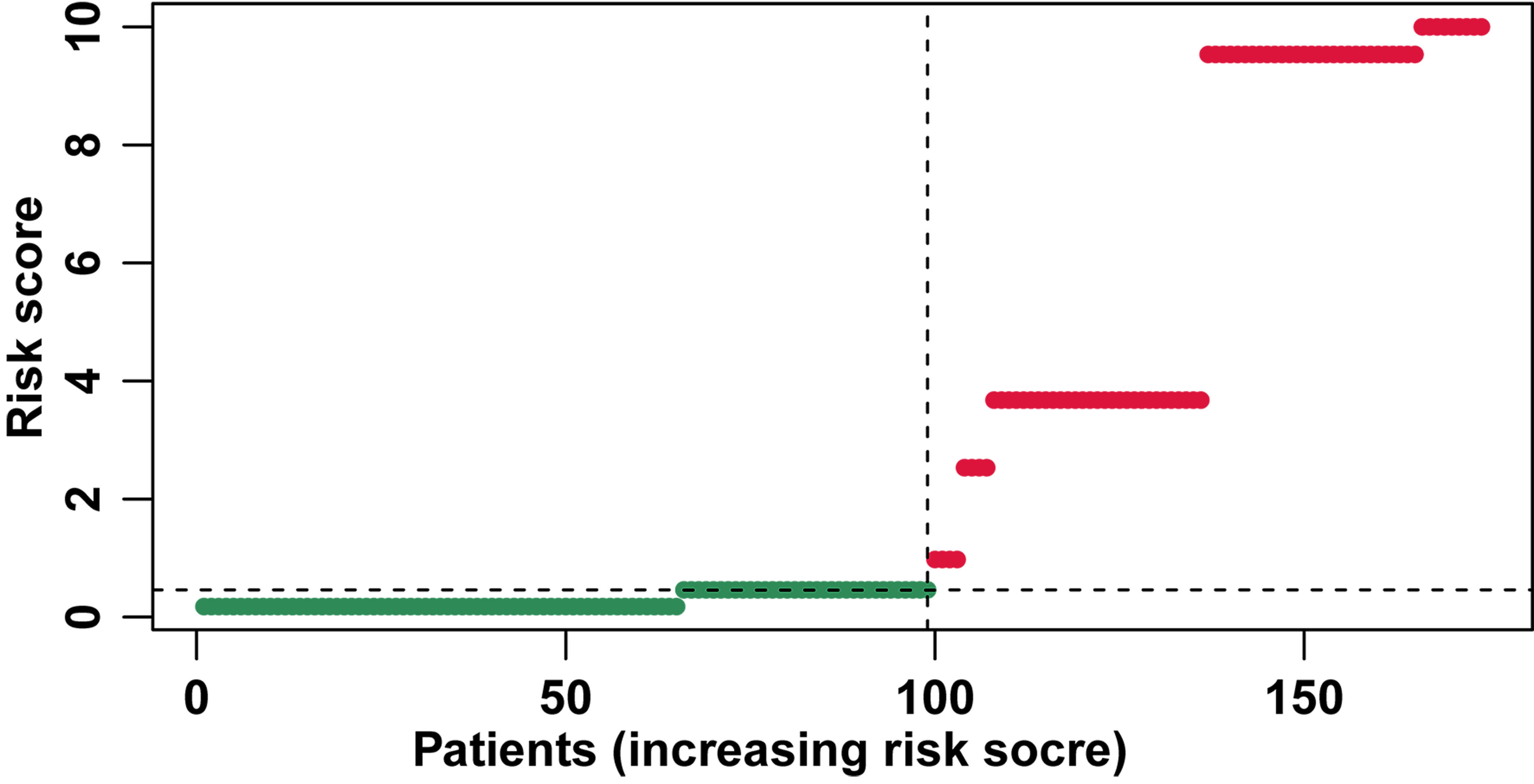

Supplement: Supplementary file 1 — Figure S1 [file CAM4-11-3407-s003.jpg]

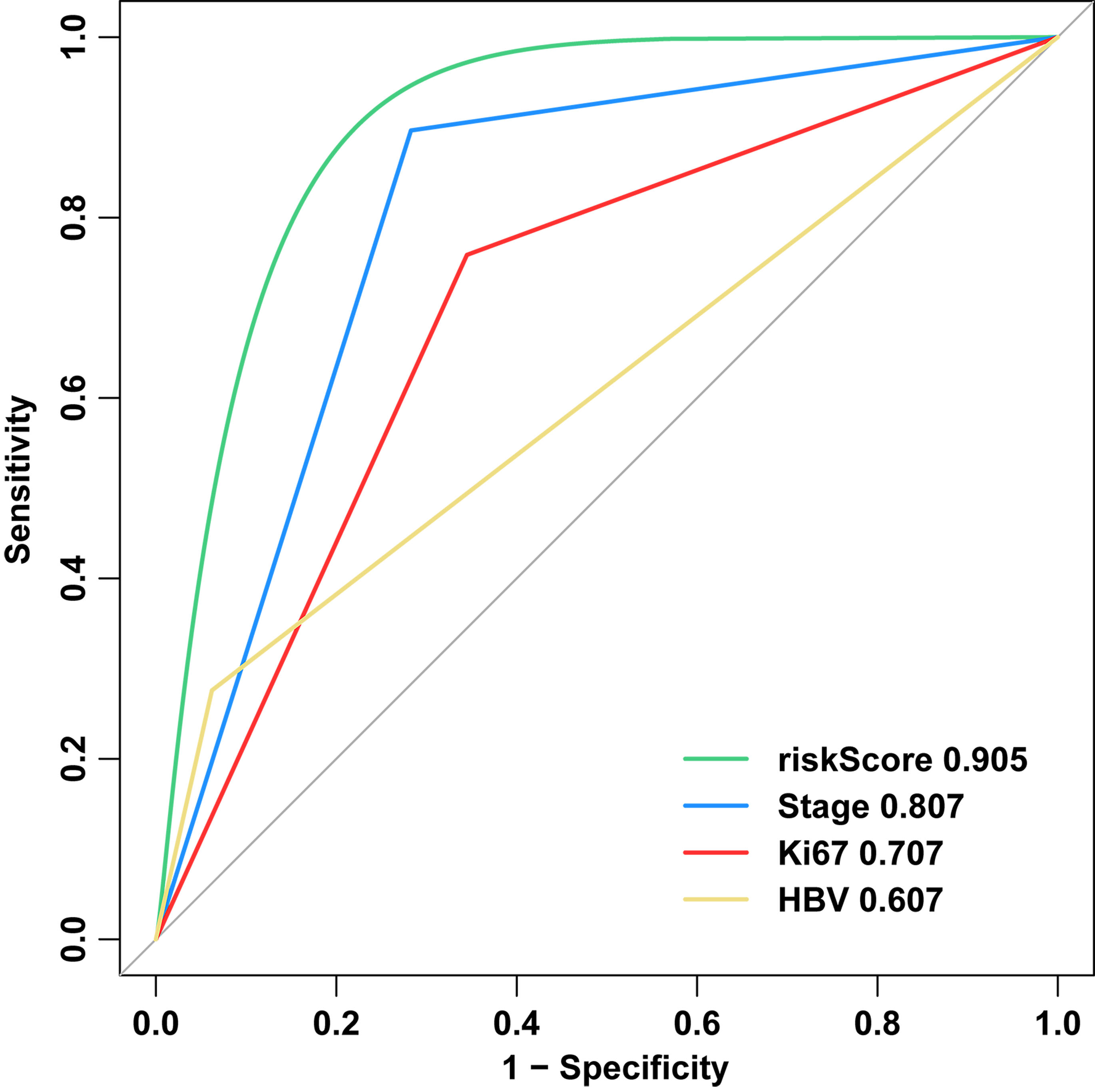

Supplement: Supplementary file 2 — Figure S2 [file CAM4-11-3407-s001.jpg]

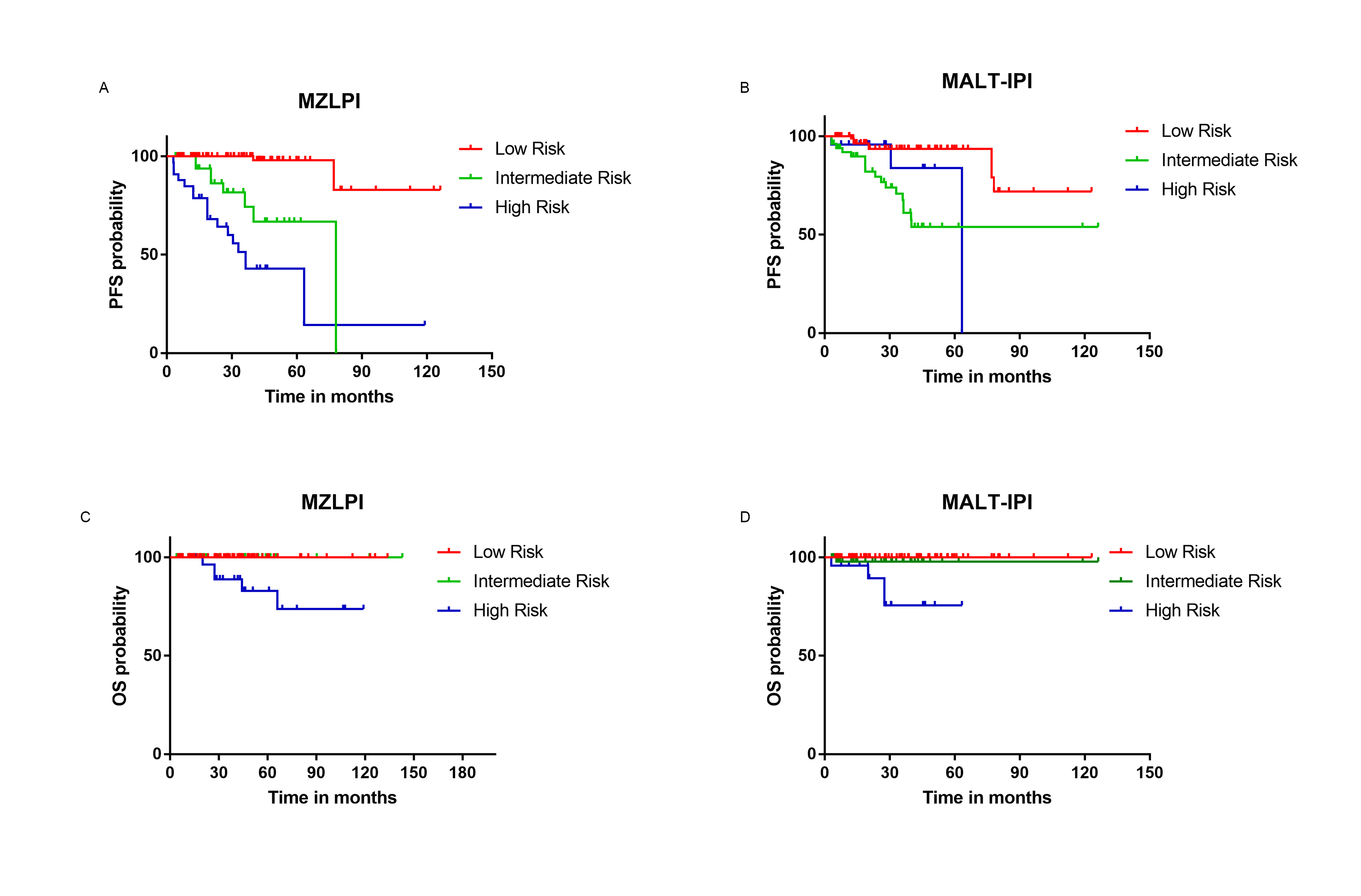

Supplement: Supplementary file 3 — Figure S3 [file CAM4-11-3407-s002.tif]
